# Blind measurements did not confirm effects of forest fragmentation on fluctuating asymmetry of a tropical butterfly *Morpho helenor*

**DOI:** 10.1007/s00114-024-01913-9

**Published:** 2024-04-23

**Authors:** Mikhail V. Kozlov

**Affiliations:** Department of Biology, University of Turku, Turku, 20014 Finland

**Keywords:** Blind measurements, Confirmation bias, Environmental stress, Reproducibility, Wing size

## Abstract

Re-evaluation of photographs of the tropical butterfly Morpho helenor from a previous study (Pignataro et al. 2023) revealed that its conclusion regarding increased wing fluctuating asymmetry in forest edge habitats compared to forest interior habitats could not be replicated. This discrepancy likely arises from (i) original measurements not being conducted blindly, (ii) insufficient photograph quality hindering accurate landmark selection, and (iii) a lack of detailed description of the measurement protocol. The likelihood of false positive discoveries within the published data concerning the impacts of environmental stress on the fluctuating asymmetry of plants and animals is probably higher than previously assumed.

**Supplementary Information:**

The online version contains supplementary material available at 10.1007/s00114-024-01913-9.

Studies addressing the impacts of environmental stressors on fluctuating asymmetry (FA; small, non-directional deviations from perfect symmetry in morphological traits of living beings) are particularly prone to confirmation bias (Kozlov and Zvereva 2015). False discoveries of the expected patterns arising from the tendency of humans to seek out evidence in a manner that confirms their hypotheses and beliefs (Rosenthal 1976) can be avoided by blinding the measurer with respect to the sample origin and/or hypothesis tested (Forstmeier et al. 2017). Regrettably, many researchers (including Pignataro et al. 2023) did not use blinding and thus made their conclusions vulnerable to criticism.

Pignataro et al. (2023) reported that FA of both length and width of forewings (but not of hindwing ocelli) of a tropical butterfly, *Morpho helenor* (Nymphalidae), in stressful forest edge habitats is greater than in benign forest interior habitats. This finding is consistent with a widely accepted (albeit insufficiently supported: Kozlov 2017; Gavrikov et al. 2023) opinion that FA of plants and animals always increases in response to stress. Here, I test the hypothesis that the between-habitat differences in FA reported by Pignataro et al. (2023) emerged due to confirmation bias.

The wing images provided by T. Pignataro were coded with random numbers, and my measurements were therefore blind. The distances between landmarks (Supplementary Material 1) were measured by a ruler in the Adobe PhotoShop 2020 program. FA calculation and data analysis follow Gavrikov et al. (2023).

The comparison between wing images and their size (as reported by T. Pignataro) revealed that data from wings with missing landmarks (Fig. S1) are not actual measurements but approximations (Supplementary Material 1). This finding questions both the quality and reproducibility of the data by Pignataro et al. (2023); I excluded the specimens with missing landmarks from my analyses.

The differences between the two independent measurements of length and width of forewing (Supplementary Material 2) appeared 3.5 to 3.7 times greater than between the measurements of a ruler from the same images (Figure S2a-c). Thus, the quality of the wing images does not allow precise positioning of the selected landmarks. Nevertheless, the differences between left and right wings were 1.6–1.8 times greater than the differences between two measurements of the same wing (Figure S2b-e), and the significant side × individual interactions (Table S1) confirm the existence of measurable FA in wing length and width.

Contrary to Pignataro et al. (2023), I did not find statistically significant differences between edge and interior habitats in FA of either length (mean ± SE; edge: 0.0077 ± 0.0007, *n* = 29; interior: 0.0082 ± 0.0011, *n* = 24; ANOVA of square-root transformed values: *F*_1, 51_=0.00, *p* = 0.96) or width of forewing (edge: 0.0102 ± 0.0013, *n* = 28; interior: 0.0138 ± 0.0024, *n* = 27; *F*_1, 53_=1.00, *p* = 0.32). Thus, forest fragmentation did not cause an increase in the FA of *M. helenor* in Brazil. This result opposes the prevailing paradigm but is consistent with recent reports on the absence of the effects of different stressors on the FA of several butterfly species in both natural and laboratory environments (Symanski and Redak 2021; Zverev and Kozlov 2021; Shkurikhin et al. 2003), as well as on the FA of ocelli in hindwings of the same individuals of *M. helenor* (Pignataro et al. 2023).

The critical examination of data by Pignataro et al. (2023) once again demonstrated that obtaining unbiased, high-precision repeated measurements needed to reliably quantify FA requires (i) blinding the measurer(s), (ii) selection of landmarks, positions of which can be identified with high accuracy, and (iii) a detailed description of the measurement protocol. My findings suggest that the proportion of false positive discoveries among the published data on environmental stress impacts on the FA of plants and animals is likely greater than currently thought.

### Electronic supplementary material

Below is the link to the electronic supplementary material.

Supplementary Material 1

## Data Availability

Original measurements are included in supplementary materials. For wing images contact the authors of the commented study.

## References

[CR1] Forstmeier W, Wagenmakers EJ, Parker TH (2017). Detecting and avoiding likely false-positive findings—a practical guide. Biol Rev.

[CR2] Gavrikov DE, Zverev V, Rachenko MA (2023). Experimental evidence questions the relationship between stress and fluctuating asymmetry in plants. Symmetry.

[CR4] Kozlov MV (2017). Plant studies on fluctuating asymmetry in Russia: mythology and methodology. Russ J Ecol.

[CR3] Kozlov MV, Zvereva EL (2015). Confirmation bias in studies of fluctuating asymmetry. Ecol Indic.

[CR6] Pignataro T, Lourenço GM, Beirão M, Cornelissen T (2023). Wings are not perfect: increased wing asymmetry in a tropical butterfly as a response to forest fragmentation. Sci Nat.

[CR7] Rosenthal R (1976). Experimenter effects in behavioral research.

[CR8] Shkurikhin AO, Zakharova EYu, Vorobeichik EL (2023) Phenotypic variability of *Aphantopus hyperantus* and *Coenonympha arcania* (Lepidoptera: Nymphalidae) in the vicinity of the Middle Ural copper smelter. Part 1. Metal content and wing length. Russ J Ecol 54: 526–541

[CR9] Symanski C, Redak RA (2021). Does fluctuating asymmetry of wing traits capture relative environmental stress in a lepidopteran?. Ecol Evol.

[CR10] Zverev V, Kozlov MV (2021). Fluctuating asymmetry of the butterfly wing pattern does not change along an industrial pollution gradient. Symmetry.

