# Supplementary material for: Blind measurements did not confirm effects of forest fragmentation on fluctuating asymmetry of a tropical butterfly Morpho helenor
Source: Naturwissenschaften. 2024 Apr 23;111(3):27. doi: 10.1007/s00114-024-01913-9 (PMC11039497; doi:10.1007/s00114-024-01913-9)
Supplement: Supplementary file 1 — Supplementary Material 1 [file 114_2024_1913_MOESM1_ESM.pdf]

Blind measurements did not confirm effects of forest fragmentation on fluctuating asymmetry of a tropical butterfly *Morpho helenor* [1]

Mikhail V. Kozlov

Department of Biology, University of Turku, 20014 Turku, Finland

mikoz@utu.fi

ORCID: 0000-0002-9500-4244

Supplementary Material 1: Landmarks and measurements

The measurements, from which fluctuating asymmetry is calculated, could be repeated with high accuracy only when they are based on precisely defined landmarks. Regrettably, Pignataro et al. (2023) did not provide a verbal description of landmarks used to measure wing length and width, and the illustration published by these authors is somewhat ambiguous. Therefore, as an additional data source, I used a short description of the protocol and a figure showing landmark positions on the butterfly wings on p. 47 of the Dissertação de Mestrado by T. Pignataro (https://repositorio.ufmg.br/bitstream/1843/39242/1/PPGEcologia_ThaisNeresGuimaraesPignataro_DissertacaoMESTRADO.pdf; accessed 10 February 2024).

The authors of the commented paper refused to supply me with a detailed protocol for wing measurements, despite the assistance of Matthias Waltert, Editor-in-Chief of The Science of Nature. Therefore I was forced to reconstruct this measurement protocol by comparing the data by Pignataro et al. (2023) with the respective wing images. For example, my measurements of wing length in the specimen coded ‘edge 1’ differ from the measurements by Pignataro et al. (2023, and pers. comm.) by 0.12–0.15 mm only. This difference is four times smaller than the mean absolute value of the difference in the length of right and left

[1] This is a comment to Pignataro et al. (2023) Wings are not perfect: increased wing asymmetry in a tropical butterfly as a response to forest fragmentation. **The Science of Nature**, 110: Art. 28.

wings across all 60 specimens of *Morpho helenor* studied by Pignataro et al. (2023) and is therefore unlikely to affect FA values calculated from these measurements.

At the same time, I surprisingly discovered that many of the data from Pignataro et al. (2023) are not actual measurements but estimates (approximations). For example, the length of right and left wings reported by Pignataro et al. (2023, and pers. comm.) for the specimen coded ‘edge 2’ exceeds the maximum distance between wing base and apex (as shown on an image) by 0.66 and 1.44 mm. To obtain these large values, the measurer should have selected the basal landmark outside the wing image. This action is neither reported nor illustrated by Pignataro et al. (2023): figure 1 in their work shows that the basal landmark in the right wing is placed at the broken wing margin rather than outside the wing image.

The placement of landmarks outside the wing image applied by Pignataro et al. (2023) is particularly uncertain in wings with damaged apical and/or basal parts (Fig. S1). For example, the length of the left wing of the specimen coded ‘interior 10’ is reported by Pignataro et al. (2023, and pers. comm.) as 58.87 mm, whereas the maximum wing size that could be measured from this image (Fig. S1c) is 54.85 mm. The difference between these values (4.02 mm) is eight-fold greater than the mean absolute value of the difference in the length of the right and left wings reported by Pignataro et al. (2023). Needless to say, the placement of a landmark outside the measured structure is subjective and therefore likely to enhance the impact of confirmation bias on the conclusions of a study.

To avoid the need to use approximations instead of actual measurements, I defined forewing length as the maximum distance between the base of the Sc stem and the external wing margin at the apex, whereas wing width was measured as the distance between the base and apex of the A1+A2 stem. The bases of these stems were defined as the points of their articulation with the 2nd and 3rd axillary sclerites, respectively. The positions of these landmarks are close to the landmarks used by Pignataro et al. (2023), and I therefore believe that both sets of landmarks are equally suitable to quantify wing FA.

The bases of both Sc and A1+A2 stems in many of the analyzed images could be identified due to an abrupt change of colour between the shining vein and the matte background. If this change was not clearly visible, then I positioned the basal landmarks at the level of wing articulation to thorax, as identified from the shape of wing margins, at about 1/3 and 2/3

distance between wing margins for Sc and A1+A2 stems, respectively. The distal landmark for wing length measurements was selected near the wing apex by moving the measurement point around the external wing margin and recording the maximum value of the distance from the basal landmark. The distal landmark for wing width measurements was selected at the point of A1+A2 arrival at the external wing margin. If this point was not clearly visible, I identified this landmark by projecting visible parts of the A1+A2 vein to the external wing margin. Finally, I excluded from my analysis the specimens (Fig. S1) that, due to wing damage, lacked at least one of the landmarks identified above.

Supplementary Material 2: Original measurements

Column 1: Habitat (edge vs interior).
Column 2: Individual (1 to 30 within each habitat; numbering follows Pignataro et al. 2023).
Column 3: Length of right forewing (pixels), measurement 1.
Column 4: Width of right forewing (pixels), measurement 1.
Column 5: Length of left forewing (pixels), measurement 1.
Column 6: Width of left forewing (pixels), measurement 1.
Column 7: Length of 50-mm scale (pixels), measurement 1.
Column 8: Length of right forewing (pixels), measurement 2.
Column 9: Width of right forewing (pixels), measurement 2.
Column 10: Length of left forewing (pixels), measurement 2.
Column 11: Width of left forewing (pixels), measurement 2.
Column 12: Length of 50-mm scale (pixels), measurement 2.

Missing value: one of the two landmarks is missing due to absence of a part of either wing apex or wing base (Fig. 1S).

```
Edge 1 460 312 458 311 395 459 313 452 311 396
Edge 2 474 321 477 331 395 473 324 474 328 396
Edge 3 461 329 466 329 396 465 325 470 335 396
Edge 4 457 311 457 314 396 457 313 456 311 396
Edge 5 487 340 483 334 397 486 341 484 332 395
Edge 6 498 361 500 349 397 502 358 495 350 395
Edge 7 492 356 489 352 396 494 353 490 354 396
Edge 8 500 343 498 348 396 498 342 493 343 396
Edge 9 462 327 456 326 397 463 329 453 326 396
Edge 10 489 344 484 339 397 491 339 482 345 396
Edge 11 505 353 510 359 397 509 361 506 358 396
Edge 12 497 343 495 339 397 498 342 488 333 396
```

```
Edge 13 486 350 483 347 397 488 345 482 345 396
Edge 14 493 337 487 336 397 489 338 487 334 396
Edge 15 489 . . 349 396 484 . . 349 396
Edge 16 468 324 459 324 396 463 324 466 326 396
Edge 17 474 331 477 331 397 476 332 477 331 396
Edge 18 497 358 497 356 397 500 358 496 353 396
Edge 19 512 354 510 355 396 514 355 510 358 394
Edge 20 492 340 486 333 396 491 338 488 337 394
Edge 21 442 304 439 309 396 443 300 441 305 395
Edge 22 490 . 485 343 396 489 . 488 343 395
Edge 23 472 344 479 341 398 479 338 478 340 396
Edge 24 468 330 463 331 398 466 327 463 329 396
Edge 25 497 341 493 346 396 492 347 490 344 396
Edge 26 474 345 473 342 396 476 346 472 348 396
Edge 27 510 347 501 347 396 504 346 502 347 396
Edge 28 474 337 475 331 396 471 335 475 333 396
Edge 29 500 349 501 344 396 498 342 495 344 397
Edge 30 498 351 496 351 396 498 349 499 350 397
Interior 1 480 348 478 334 397 477 346 474 332 397
Interior 2 506 359 500 356 397 503 360 501 357 397
Interior 3 489 340 492 343 397 490 336 490 346 396
Interior 4 466 321 . . 397 463 329 . . 396
Interior 5 495 343 . 346 396 498 345 . 344 397
Interior 6 517 369 527 369 396 516 367 528 371 397
Interior 7 473 325 472 333 396 473 330 471 334 396
Interior 8 488 340 487 335 396 488 341 488 341 396
Interior 9 470 329 477 345 396 474 328 484 343 397
Interior 10 461 318 . 314 396 457 313 . 314 397
Interior 11 501 342 493 341 397 498 341 493 343 397
Interior 12 423 288 420 288 363 421 292 420 288 363
Interior 13 . . 500 349 396 . . 504 347 396
Interior 14 479 352 483 349 396 477 352 476 352 396
Interior 15 453 324 450 327 372 452 327 448 327 372
Interior 16 464 319 . 308 395 465 319 . 307 397
Interior 17 495 340 493 343 396 497 337 493 345 397
Interior 18 453 315 460 319 396 452 313 459 323 397
Interior 19 445 320 453 332 396 443 321 450 334 397
Interior 20 510 359 505 361 396 502 362 502 360 397
Interior 21 511 361 515 358 396 514 357 515 356 396
Interior 22 474 346 476 345 396 478 343 475 342 396
Interior 23 456 313 459 312 368 459 312 456 311 367
Interior 24 482 343 494 348 396 487 345 492 348 396
Interior 25 485 333 483 334 396 485 333 488 334 396
Interior 26 348 . 350 249 272 348 . 350 249 272
Interior 27 338 248 341 243 272 339 249 342 246 272
Interior 28 503 344 499 342 397 505 337 503 342 397
Interior 29 489 343 489 349 397 489 342 492 350 397
Interior 30 504 357 . 358 396 505 356 . 356 396
```

Table S1. Basic statistics on repeated blind measurements of the left and right forewings of *Morpho helenor*.

Trait	Directional asymmetry, mm	Source of variation		ME5 [1]
		Side	Side × individual	
Length	-0.03	$F_{1, 52} = 1.62$, $p = 0.21$	$F_{52, 113} = 3.10$, $p < 0.0001$	0.51
Width	-0.15	$F_{1, 54} = 0.04$, $p = 0.84$	$F_{54, 115} = 5.53$, $p = 0.0001$	0.69

[1] ME5 = (MSi – MSm)/(MSi + MSm), where MSi and MSm are the interaction and error mean squares from a side × individual ANOVA for two measurements of each side in each leaf. This index expresses FA variation as a proportion of the total variation between sides, which includes variations due to both FA and measurement error (Palmer and Strobeck 2003).

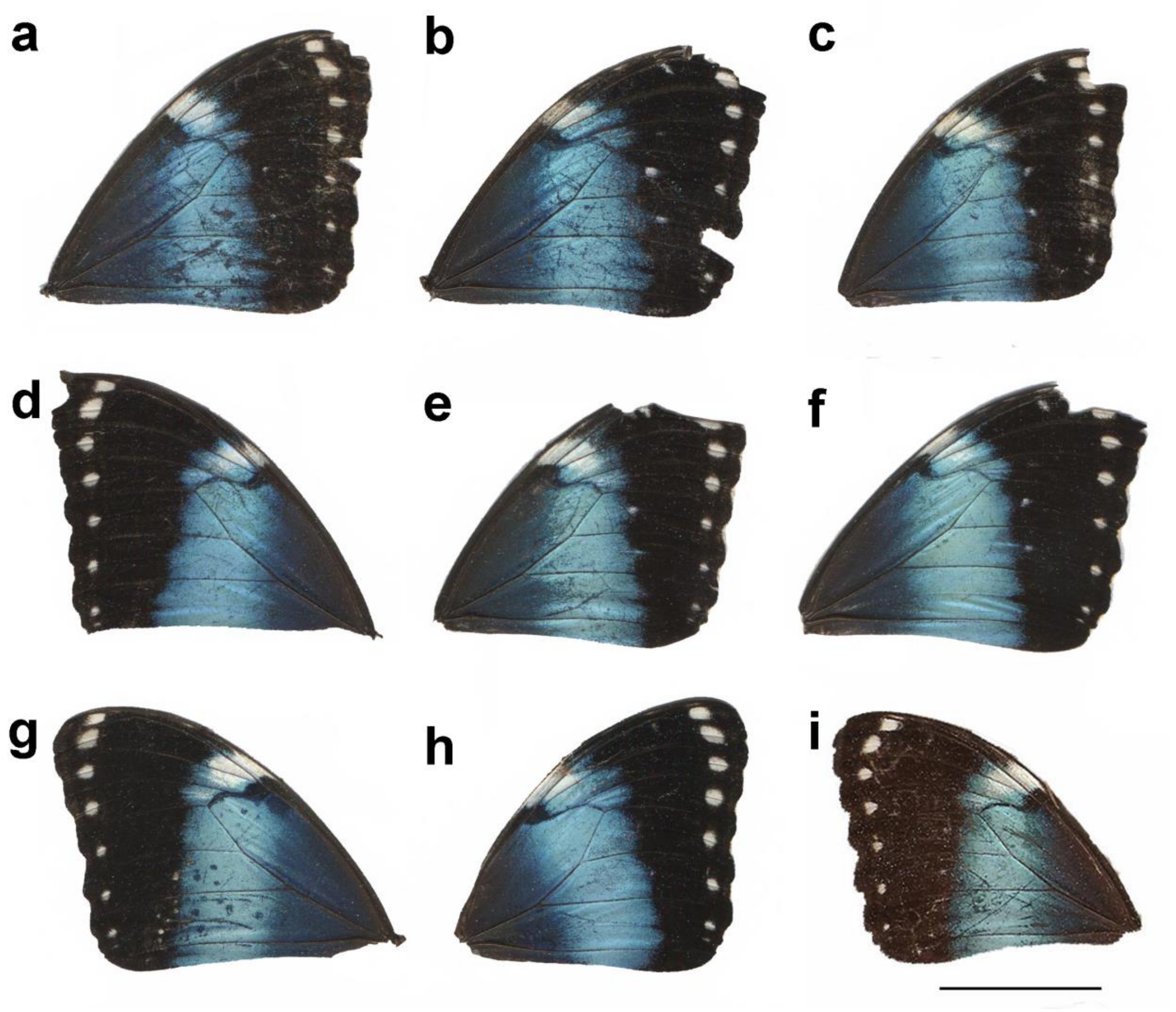

Fig. S1. Fore wings of *Morpho helenor* specimens, which have been excluded from the analyses due to impossibility to precisely measure their length or width: **a**, edge habitat #15 (apex missed); **b**, interior habitat #5 (apex missed); **c**, interior habitat #10 (apex missed); **d**, interior habitat #13 (apex, dorsal wing margin and base missed); **e**, interior habitat #16 (apex missed); **f**, interior habitat #30 (apex missed); **g**, edge habitat #22 (base missed); **h**, interior habitat #4 (base missed); **i**, interior habitat #26 (base missed). Scale: 25 mm. Photograph courtesy of T. Pignataro.

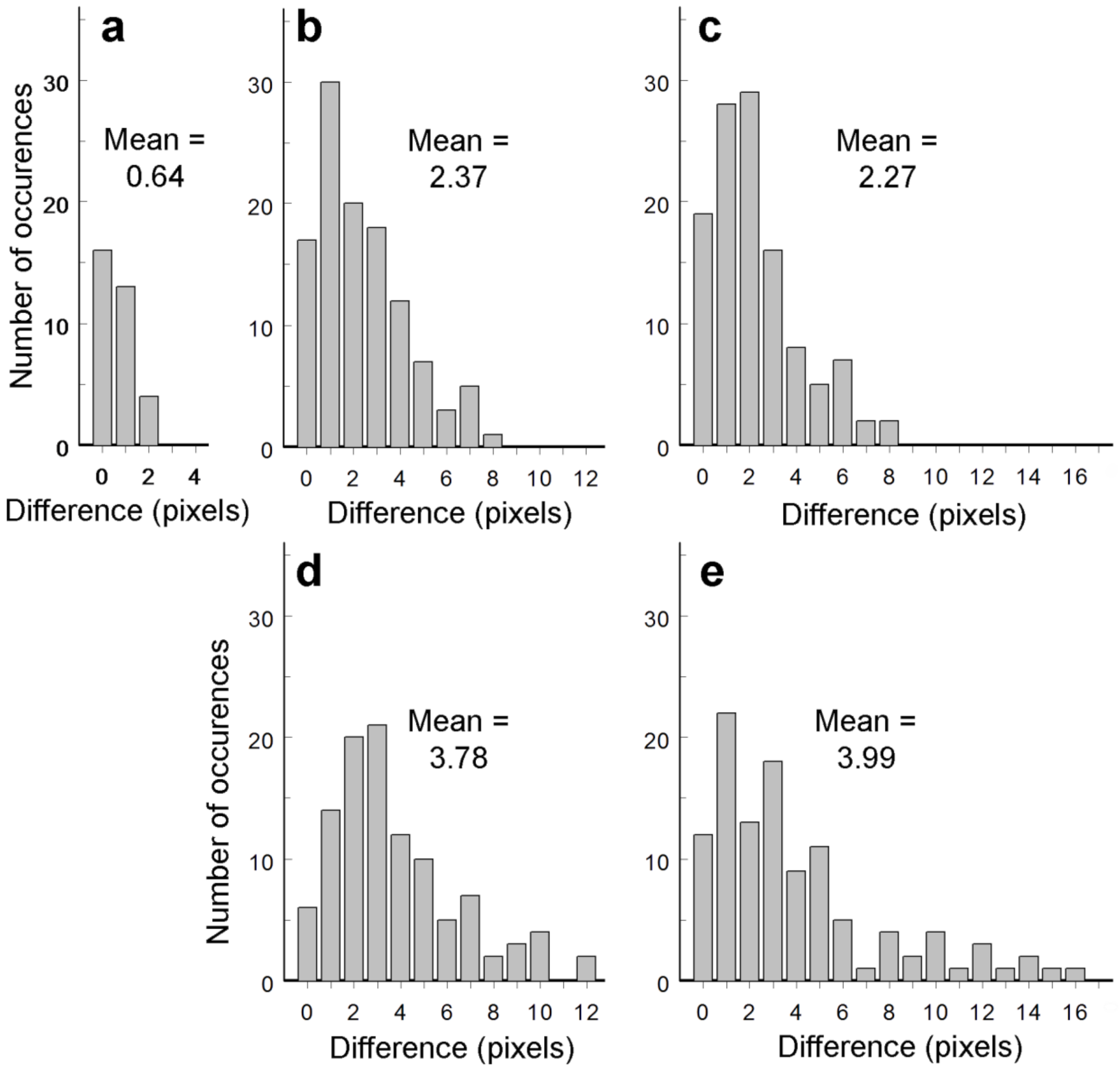

Fig. S2. Distribution of the absolute differences between two independent measurements of 50-mm scale (a), forewing length (b) and width (c), and between length (d) and width (e) of left and right forewings of *Morpho helenor*. Differences in absolute accuracy of measurements between scale (a) and wing length (b): Kruskal-Wallis test, $\chi^2_1 = 26.9$, $p < 0.0001$; between scale (a) and wing width (c): $\chi^2_1 = 25.9$, $p < 0.0001$; between wing length (b) and asymmetry in wing length (d): $\chi^2_1 = 14.5$, $p < 0.0001$; between wing width (c) and asymmetry in wing width (e): $\chi^2_1 = 11.1$, $p = 0.0009$.
